# Potential Clinical Application of Organs-on-a-Chip in Periodontal Diseases: A Systematic Review of In Vitro Studies

**DOI:** 10.3390/dj11070158

**Published:** 2023-06-26

**Authors:** Carlos M. Ardila, Gustavo A. Jiménez-Arbeláez, Annie Marcela Vivares-Builes

**Affiliations:** 1 Basic Studies Department, School of Dentistry, Universidad de Antioquia UdeA, Medellín 050010, Colombia; 2 School of Dentistry, Institución Universitaria Visión de Las Américas, Medellín 050031, Colombia; gustavo.jimenez@uam.edu.co (G.A.J.-A.); anny.vivares@uam.edu.co (A.M.V.-B.)

**Keywords:** organ-on-a-chip devices, microphysiological systems, organotypic cell cultures, periodontal diseases, in vitro techniques

## Abstract

The periodontium is a unique organ from the standpoint of building an organ-on-a-chip (OoC) since it is a system that is continually threatened by microorganisms, their noxious compounds, and antigenic components. At the same time, periodontal health depends on a balanced connection between the host and the bacteria in the oral cavity, which is a complex micro-ecological environment. The objective of this systematic review of in vitro studies is to revise the potential clinical application of OoC in periodontal diseases. PRISMA was used to guide this analysis. The review framework made use of several databases, including SCOPUS, PubMed/MEDLINE, SCIELO, and LILACS as well as the gray literature. This systematic review comprised seven studies. The clinical efficacy of OoC in periodontal diseases was observed in models of the gingival crevice for the research of periodontitis, periodontal medication analysis, the interaction of multiple microbial species, pH measurements in in situ-grown biofilm, testing antimicrobial reagents, evaluation of mucosal interactions with microorganisms, and a device for quantitative exploration of microorganisms. OoC has the potential to advance our understanding of periodontal diseases by providing a more accurate representation of the oral microenvironment and enabling the development of new treatments.

## 1. Introduction

Periodontitis is a chronic inflammatory disease that affects the periodontium, which includes the tissues that surround and support the teeth, such as the gums, periodontal ligament, and alveolar bone. It is characterized by the destruction of the connective tissue and bone that support the teeth, leading to tooth loss if left untreated [1]. The etiology of periodontitis is multifactorial and involves the interaction of microbial, genetic, and environmental factors. The primary cause of periodontitis is the accumulation of bacterial plaque on teeth and gums, which triggers an immune response that leads to inflammation and tissue destruction [2].

Biofilm formation is a crucial step in the pathogenesis of periodontitis. A biofilm is a complex, three-dimensional community of microorganisms that are embedded in an extracellular matrix of their own production. The biofilm protects the microorganisms from host defenses and antibiotics, allowing them to persist in the periodontal tissues and cause ongoing inflammation and tissue destruction [3,4].

In periodontitis, the initial microbial colonization on tooth surfaces is predominantly composed of Gram-positive bacteria such as Streptococcus spp., Actinomyces spp., and other facultative anaerobes. As the biofilm matures and thickens, the microorganisms shift to a more anaerobic and Gram-negative flora, including *Porphyromonas gingivalis*, *Tannerella forsythia*, and *Treponema denticola*. These species are known as periodontal pathogens and have been implicated in the pathogenesis of periodontitis [3,4,5].

The biofilm allows these microorganisms to interact and form synergistic relationships, leading to increased virulence and resistance to antimicrobial agents. Overall, the formation of a complex biofilm in periodontitis is a critical factor in the development and progression of the disease, and understanding the role of specific microorganisms in biofilm formation may lead to new strategies for preventing and treating periodontitis [6].

Invasion of the gingival epithelium and the connective tissue by bacteria and their metabolites can activate periodontal cells, encourage the production of cytokines and polymorphic granulocytes, and cause the secretion of a variety of pro-inflammatory mediators [7,8]. The gingival epithelium plays a crucial role in the preservation of periodontal tissue homeostasis by serving as a structural barrier between the underlying tissue and the external environment [7,8,9]. It actively contributes to the body’s defense against infection in addition to acting as a physical barrier. The gingival epithelium further increases host responses and integrates innate and acquired immunity in the innate host immune defense response by interactions of epithelial cells with bacteria and metabolites [10,11]. The illness is complicated by tooth migration, drifting, hypermobility, and even loss once the junction epithelium and periodontal pocket wall integrity are lost, which worsens the condition and lowers the quality of life for those who are affected [12,13]. Moreover, a lot of evidence suggests that nearby bacteria, their byproducts, and even inflammatory mediators produced by inflamed periodontium could spread throughout the body through hematogenous dissemination and increase the risk of several systemic diseases, such as atherosclerosis, poor pregnancy outcomes, rheumatoid arthritis, aspiration pneumonia, and cancer [14,15]. To construct the ecological network in terms of periodontal tissue form and function as well as to advance our understanding of host–pathogenic factors and therapeutic medications, research on biofilm and the gingival epithelial barrier is advantageous [16,17].

Most investigations use monolayer-grown gingival and periodontal cells in static culture plates and then expose the cells to plaque microorganisms or their substitutes to study the interactions between the periodontal host and microbiome [16,18]. In these reductionistic systems, the host cells’ culture media is directly infused with the microorganisms or their substitutes. The complexity of host-microbiome interactions has been increased because of the use of 3D organotypic models of gingival tissue equivalents cultivated within static Transwell culture inserts [19,20]. The apical surface of the tissue analogs is often where the bacteria or their substitutes are inserted in these 3D culture systems. However, these 3D static culture models and monolayer models are unable to accurately capture the natural milieu of the gingival crevice and the pathophysiology of periodontal disease. It has been indicated that the amount of time that can be spent studying the interactions between the host and oral microbes in research involving the co-culture of human cells and live microorganisms is frequently limited because the accumulation of harmful by-products from microbial metabolism causes harm to the host cells [21,22].

The above usually limits the interaction between living oral microbiota and host cells to brief intervals [23,24]. It is also common to use attenuated bacteria or their substitutes, such as Toll-like receptor agonists or virulence factors like lipopolysaccharide and lipoteichoic acid [25,26]. Moreover, gingival crevicular fluid and its flow’s influence on host–microbe interactions have so far been disregarded. The development of a persistent symbiotic interaction between the plaque microbiota and gingival crevicular tissue within a dynamic environment drenched in the gingival crevicular fluid is essential for understanding the onset and progression of periodontal disease [16]. On the other hand, the preferred strategy for mimicking and forecasting the response of periodontal tissues to medications, infections, and environmental contaminants has long been animal models. However, due to the non-human nature, animal studies are expensive, time-consuming, and contentious [17]. Furthermore, animal models cannot accurately reproduce the exact human physiology. Despite ongoing advancements in two-dimensional cell culture technologies, cell culture assays still lack the complexity of biological systems due to their simple cell types. They are unable to communicate between organs or between tissues, and they are also unable to foresee complex host immunological responses or the impact of metabolite action on tissues other than the targets [27]. These limitations highlight the need for novel techniques to develop physiologically accurate in vitro models [16,17].

Microfluidic cell culture technology, often known as organ-on-a-chip, is developing quickly and has great promise for creating in vitro models since it can more accurately mimic the essential structural and functional characteristics of human tissues and organs. Multiple cell interactions may be made possible by organs on a chip technology, making them more physiologically applicable [28]. Even though most of the existing organ-on-a-chip system cannot be organs, they may be able to partially emulate the microarchitectures of the functional units of organs or tissue–tissue interfaces. They might also combine different mechanical and biological stimuli to create functional artificial engineering organs. Typically, microfluidics, biomimetics, and biological microelectromechanical systems (bioMEMS) can be used with organs on a chip system [27]. These innovative platforms could connect several organs and investigate their interactions on a single chip thanks to advancements in microengineering technologies with microfluidic controls [29]. The construction of gut [30] and lung [31] on-chip models using such microfluidic devices has been successful, and research exploring protracted host–microbial interactions under dynamic fluid flow and mechanical strain has been carried out. This, the use of an organ-on-a-chip microfluidic system, such as oral mucosa, biofilm, gingival epithelium, and gingival crevices, in periodontal research is currently attracting attention.

The periodontium is a unique organ from the standpoint of building an organ-on-a-chip since it is a system that is continually threatened by microorganisms, their noxious compounds, and antigenic components [16,17]. At the same time, periodontal health depends on a balanced connection between the host and the bacteria in the oral cavity, which is a complex micro-ecological environment [16]. The ability of a model to replicate the essential physiological and biological traits of its in vivo prototype determines how effective the model is [16,17].

Some narrative reviews have explored the organ-on-a-chip application in the oral cavity [31,32,33]; however, there is no known systematic review that explores this technology in periodontics. Therefore, the objective of this systematic review is to revise the potential clinical application of organs on a chip in periodontal diseases.

## 2. Materials and Methods

### 2.1. Search Strategy

PRISMA (Preferred Reporting Items for Systematic Reviews and Meta-analyses) was used to guide this analysis [34]. The review structure made use of several databases, including SCOPUS, PubMed/MEDLINE, SCIELO, and LILACS as well as the gray literature. The terms organ-on-a-chip, lab-on-a-chip, microphysiological systems, microfluidics, bioassays, periodontal soft tissue, periodontitis, periodontal diseases, biofilm, and studies published in all languages were included in searches that were carried out using keywords and MeSH terms up until April 2023. Using Boolean operators (AND, OR), the next exploration method was used to research databases: “organ on a chip” OR “lab on a chip” OR “microphysiological systems” OR “microfluidics” OR “bioassays” AND “periodontal diseases” AND “periodontitis” AND “periodontal soft tissue” AND “biofilm”.

### 2.2. Selection Criteria

The publications that were considered for inclusion in this review met the following criteria: usage of microfluidic platforms or lab-on-a-chip, application of 3D printing, and/or bioprinting techniques in the context of organs-on-a-chip devices.

Abstracts, reviews, systematic reviews, or meta-analyses were excluded, as well as publications that only used computational methods, brief communications, conference articles, patents, case reports, and studies that lacked key details on the manufacturing process.

### 2.3. Question

The subject of this systematic review is: in experimental periodontal disease, what potential clinical applicability of organs on a chip exists? 

P: Experimental periodontal disease.

I: Organs on a chip.

C: Control experiments.

O: Potential clinical applicability.

### 2.4. Review Process

Two researchers (CMA and AV) reviewed the titles and abstracts to select papers that might be eligible. Given the possibility of disagreement in the choice of studies, a third author (GJ) could intervene. Utilizing the statistical test Kappa, the significance of observer agreement (>92) was assessed.

### 2.5. Data Collection

A table was created using the selected studies’ most important data. This process was carried out independently by each researcher. The data were compared after that. The information included the names of the authors, the date the work was published, the application of the organ-on-a-chip in periodontics, and details about the most important features of an organ-on-a-chip device, such as the materials used in its construction, chip design, cell type, tissues, microorganisms and culture, and the main results. 

### 2.6. Risk of Bias

Using a previously developed scale [35], two authors assessed the quality and risk of bias of the studies that were included. The tool used makes it possible to evaluate the study through 15 items: design (objective, sample, baseline characteristics, and co-interventions), the measures studied (measurement method, blinding examiner, statistician, described reliability, and level of agreement), statistical analysis (appropriate analysis, co-interventions, subgroup analysis, statistical significance, and confidence intervals), and clinical significance.

## 3. Results

**Figure 1:**
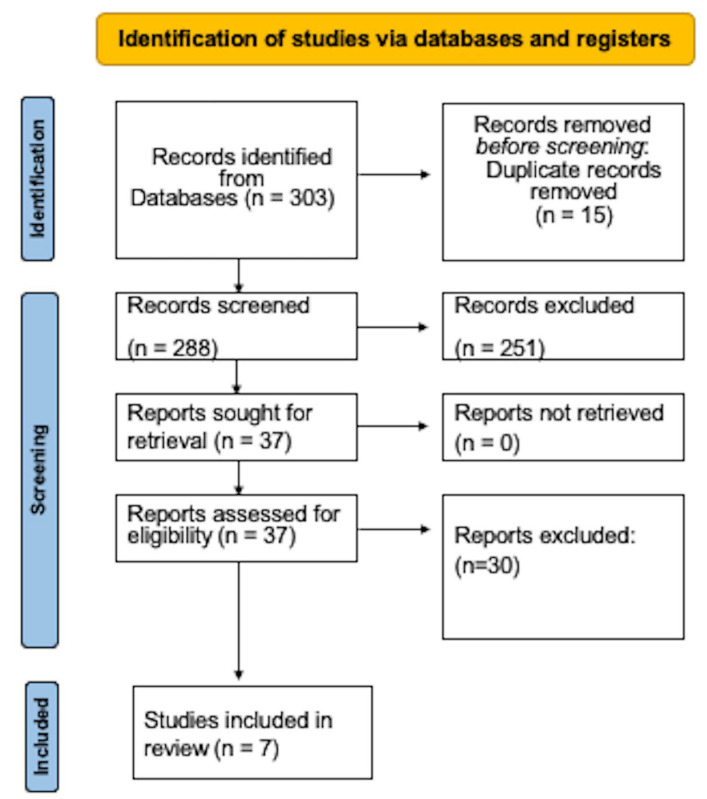
Process flowchart for choosing the studies.

There were 303 reports in the electronic exploration. A total of 251 articles were disregarded, and 15 duplicate publications were also omitted. Thirty other investigations were left out after reading the entire text since they did not fit certain requirements. Finally, this systematic review comprised seven studies [16,17,36,37,38,39,40] (Figure 1).

Table 1 displays the characteristics of the included studies. The studies in question were published between 2016 [40] and 2023 [16].

As seen in Table 1, this systematic review showed that the organ on a chip has the following potential clinical applications in periodontal diseases: a model of the gingival crevice for the research of periodontitis [16], a periodontal device for medication analysis and for functional studies [17], a device for screening the interaction of multiple microbial species at microscale [36], a model for pH measurements in in situ-grown biofilm [37], a device for testing antimicrobial reagents [38], a model to evaluate mucosal interactions with microorganisms and the responses of epithelial and subepithelial layers [39], and a device for high-throughput and quantitative exploration of dental microorganisms under diverse combinations of micro-environmental elements [40].

As can be seen in Table 1, all these models showed efficacy in each of the areas considered. Under near-microphysiological settings, the proposed in vitro platform can research complex host–microbe interactions in periodontal disease and the development of periodontal therapies [16]. The planned epithelium–capillary interface system has been shown to be a suitable model for researching periodontal soft tissue function and medication administration [17]. The findings confirmed a scenario in which the interaction of specific species in proximity causes the creation of an exclusion zone [36]. By comparing pH developments in in situ grown dental biofilms under static and dynamic settings, it was established that medium flow had a profound impact on metabolic activities in dental biofilms [37]. The biofilm on-chip has been demonstrated to be capable of measuring changes in biofilm architecture as well as detecting potential antimicrobial and anti-biofilm effects of candidate drugs [38]. With a histology-like view of the tissue layers, the oral mucosa-on-a-chip is ideal for examining oral mucosal interactions with bacteria and biomaterials [39]. Finally, the microfluidic device can test biofilm qualities under various growth conditions such as seeding bacteria populations, growth medium compositions, medium flow rates, and dissolved gas levels [40].

Table 1 also depicts that the creation of an organ-on-a-chip can be accomplished using a variety of materials and microfabrication techniques. Thus, nanometer-scale chip features can be manufactured into silicon wafers using photolithography. Reservoirs, chambers, and microchannels are examples of compartments that are frequently seen in microfluidic chips. Furthermore, functional parts that are designed to transport the liquid in a specific way include valves, mixers, and pumps. As found in this review, the construction of the organ on a chip is accomplished primarily in lab settings using polydimethylsiloxane silicone rubber.

The design of the chips used in the models studied in this review included the one-chamber [37,38], the parallel-chamber [16,17,39], and the multiarray [36,40].

The tissues, cells, and microorganisms studied in the investigations explored in the current review included epithelium and connective tissue of the gingival crevice, gingival fibroblasts, keratinocytes, saliva with and without cells, and *Porphyromonas gingivalis*, *Fusobacterium nucleatum*, and species *of Streptococcus* and *Actinomycetaceae*, among others. 

The different culture media used in the modeling of the organ-on-a-chip are also presented in Table 1. These include colonization of commensal and pathogenic bacteria [16,36,40], inoculation of vascular endothelial cells and gingival epithelial cells [17], biofilm collection [37,38], and culture of gingival keratinocytes and fibroblasts [39].

All studies reviewed had a low level of risk (Table 2). However, it is important to note that all the models had different objectives, conceptualization, and methodology, as well as different outcome variables. All this makes it difficult to carry out joint quantitative assessments.

## 4. Discussion

This first systematic review of organs-on-a-chip in periodontal diseases presents their potential clinical applications. Organ functionality can be reproduced in vitro using organ-on-a-chip models, which combine microsystem engineering, cell biology, and microfluidics. They are about to become widely used as innovative models for researching medication response and toxicity in academia and the pharmaceutical business [16,17,31]. This innovative technology has shown important advances that allow the elucidation of some complex mechanisms that occur in periodontal diseases.

The studies reviewed here showed potential clinical applications in the modeling of the gingival crevice with microbial colonization of the host under conditions of health or disease, in the characterization of the dynamic interactions of microorganisms and in the impact of stimulated saliva flow on the change of the biofilm pH, as well as in the evaluation of antimicrobial reagents. 

Microfabrication and microfluidics used to design and create an on-chip gingival crevice devices to replicate the microenvironment of the connective tissue wall of the gingival crevice were explored in this review [16,17]. This microfluidic system enables the co-culture of microorganisms (commensal and pathogenic) from the plaque microbiome and balanced three-dimensional cultivation of gingival connective tissue equivalents with replicated, unidirectional gingival crevicular fluid flow [16]. A growing body of research has focused on creating and using in vitro three-dimensional organotypic cultures of reconstructed gingival epithelium, connective tissue, and full-thickness tissues to mimic the native tissue microenvironment and comprehend the interactions between the periodontal host and microbiome [41,42,43]. In vitro organotypic models of the gingival crevice, including the dentogingival junction, sulcular epithelium, and junctional epithelium, have also been shown to form in earlier investigations [44,45]. These prototypes have shed light on the interactions between the host’s microbiome and the epithelial barrier as well as the etiology of periodontal disease caused by subgingival biofilms, *P. gingivalis*, and *F. nucleatum* [42,43]. In this context, the vascular endothelial cells’ barrier function in the periodontal soft tissue barrier cannot be disregarded [46]. Smaller substances pass through junctions more rapidly and maintain an effective endothelial barrier, as shown in this review by the human vascular endothelial cells monolayer’s easy expression of tight junctions and increased permeability to tracers with lower stokes radius [17]. Moreover, endothelial and epithelial cultures on the membranes revealed high cell viability (>90%) of human vascular endothelial cells and human gingival epithelial cells cultured in the microdevice on day 3. This was due to the much thinner culture membrane that decreased the distance between co-cultured cells from the epithelium–capillary interface model, allowing the cells to maintain a small population while simulating the function [17]. Different kinds of functional cells and perfusion media can be easily and independently recovered from this device, as opposed to animal models and conventional flat-panel models. Therefore, an offline analysis, such as immunofluorescence staining, can easily characterize a variety of biomarkers of each type of cell [17].

Research is still primarily focused on analyzing the intricate structure of microbial communities in the oral cavity and how it affects both health and disease. Physical and metabolic traits help organisms colonize different oral surfaces successfully and continue to survive in this challenging environment [47]. Herein, in this regard, microbial mono- and co-cultures of oral isolates were observed using polydimethylsiloxane microfluidic devices in nanoliter quantities [36,39,40]. When co-cultured with *Streptococcus cristatus* or *Streptococcus salivarius* but not *Streptococcus oralis* or *Streptococcus mitis*, *Actinomyces graevenitzii* microcolonies formed clear “exclusion zones” around them, according to detailed microscopy. Additionally, no exclusion zones were seen when any of the tested *Streptococcal* species were co-cultured with *Streptococcus odontolytica* or *Actinomyces naeslundii*, indicating that the phenomenon may be somewhat species-specific [36]. Exclusion zones did not form between *A. naeslundii* and *S. salivarius*, which do not co-aggregate in suspension. It is yet unknown how their formation corresponds to readouts from various co-culture methods [48]. *Actinomyces* and *Streptococcus*’s in vivo spatial arrangements suggest that these genera frequently coexist close by [49]; nevertheless, these observations only offer a limited amount of information at the species level [36].

In a co-culture with *Sprepptococci*, *F. nucleatum*, *A. naeslundii*, and *P. gingivalis*, an organ-on-a-chip also demonstrated its functionality [40]. Regarding *Streptococci*, *F. nucleatum*, and *A. neaslundii*, the coverage of these bacteria shows reasonable ratios when compared to the stated ratios for in vivo biofilms [50]. Essentially, the percentages of the main bacterial species would vary depending on how much soluble matter was present during dental biofilm formation. A larger concentration of L-arginine monohydrochloride, for instance, would enhance the *Fusobacteria* population in dental biofilms [51]. Together, the dental biofilm culture applications in microfluidics demonstrated that the microenvironments maintained by the artificial teeth device can support proper growth, viability, and bacterial compositions of dental biofilms. The artificial teeth device can also be used for studies of the co-colonization dynamics of multiple target bacterial species and the associated physiological cell–cell interactions by performing parallel cultures of multiple biofilms in the microchamber arrays with various combinations of soluble factors [40].

Moreover, this review also showed that a co-cultured microfluidic mucosal model on a chip was created to quickly evaluate mucosal remodeling as well as the responses of the epithelial and subepithelial layers to difficulties typically encountered in the oral environment. In a three-channel microfluidic device with connected pores, a gingival fibroblast-filled collagen hydrogel was created in the center channel. This was followed by a keratinocyte layer adhered to the collagen exposed in the pores. This arrangement resulted in flow-supporting apical and subepithelial side channels [39]. This microfluidic configuration permits flow in the luminal channels, which in the future can happen during culture and mimic the flow of saliva or gingival crevicular fluid, which is known to slough keratinocytes and maintain epithelial layer morphology. This contrasts with static well plate culture [52]. The longest culture duration examined was seven days; however, keratinocyte infiltration of the collagen gel and gel contraction may limit culture beyond this time. Future work will focus on adding flow, creating a collagen layer that is stiffer and/or denser, and analyzing how differentiation and inflammatory markers are expressed in response to both pathological therapies and regular culture conditions [39].

This systematic review also assessed the ability of the organ on a chip to quantify changes in biofilm architecture and identify potential antimicrobial and anti-biofilm effects of candidate medicines [38]. Using identical media and inoculum preparations, this model system successfully developed oral multi-species biofilms over a 22 h period, simulating the 48-well system. The installation of a second inlet well allowed for the automatic supply of exogenous aqueous treatment solutions to the location of biofilm formation, which is where the distinction lies. This feature makes it easier to evaluate potential formulations and their effects on biofilms than the 48-well system, which necessitates stopping biofilm growth and manually administering therapy to the solitary intake [53]. Previous research describing stannous fluoride’s antibacterial effects on important oral species [54] lends support to the idea that it changes the architecture of biofilms. In clinical investigations and in the lab, stannous fluoride dentifrices have been shown to have anti-gingivitis and anti-plaque effects on tooth plaque [55,56]. It was shown in the inquiry under investigation here that there is routine exposure of developing oral biofilms to stannous fluoride solutions at 3439 p.p.m. The amount of stannous present in dentifrice (Sn^2+^) was sufficient to impede or delay biofilm growth and change the architecture of the biofilm [38].

Finally, a flow cell with a customizable shape was created for microscopy investigation of in situ-grown biofilm samples under shear-controlled flow in another study that was assessed here. By contrasting pH changes in in situ-grown dental biofilms under static and dynamic circumstances, the influence of medium flow on metabolic activities in dental biofilms was established [37]. Since processes seen in mono-species or even multi-species laboratory biofilms cannot be easily extrapolated to a clinical setting, there is a critical need to increase research on in situ-grown biofilms [57]. Due to the unavailability of shear-controlled micro-fluidic devices that can be tailored to a specific sample shape, it is challenging to replicate the flow conditions of such real-life circumstances [58]. Many of the drawbacks of conventional flow chambers can be overcome with this newly developed 3D printed flow-cell [37]. This study’s experimental approach, as compared to earlier research, more closely matches in situ settings since it provides flow velocity and film thickness that are like those seen in the oral cavity [59,60].

As previously mentioned and as was recently reported [31], the inherent benefits of organs-on-a-chip hold promise for addressing the two main challenges in current periodontal research, including the need to mimic tissue interfaces and simulate the multifactorial oral environment. It becomes clear from trying to classify periodontal models that the function and hence the application of the chip are determined by the chip’s overall design. In this systematic review, the chips used in the included studies were the one-chamber [37,38], the parallel-chamber [16,17,39], and the multiarray [36,40]. The simplest design is the one-chamber chip, which consists of a single culture chamber connected to channels for fluid transfer. This design enables the simulation of various mechanical oral environments. In the oral environment, for example, the mechanical stress brought on by saliva flow is unavoidable and closely tied to the development and properties of a biofilm [31,37,38]. To study pathophysiological processes, the parallel-chamber chip is primarily employed as a scaffold to replicate real tissue architecture. This construction connects two or more parallel chambers either vertically or horizontally using a variety of interstitial elements, such as tubes, holes, and membranes [16,17,31,39]. Lastly, a matrix of identically sized chambers connected by channels makes up a multiarray chip. The chambers serve the same purpose as the wells for cell cultivation. This design is mostly utilized for high-throughput screening since it creates various conditions in each chamber [31,36,40].

While an organ-on-a-chip has potential applications in periodontal disease research, it also has several limitations that must be addressed. The oral microbiome is complex and dynamic, and its interactions with host tissues are poorly understood. Replicating the oral microbiome in an organ-on-a-chip is challenging, and the lack of accurate representation of the oral microbiome can limit the applicability of organs on a chip in periodontal disease research [16,31]. There is currently a lack of standardized protocols for organ on a chip fabrication and operation, which can make it difficult to compare results between studies or replicate experiments. This can limit the reliability and reproducibility of organ-on-chip-based research [61,62]. Organs on chips are typically small and cannot be easily scaled up to produce large quantities of tissue or organ models. This can limit their usefulness in applications such as drug discovery, where large quantities of tissue are needed for screening [61,62,63]. Developing and maintaining can be expensive, which can limit their accessibility to researchers and companies with limited resources. Organs-on-a-chip are currently limited in their ability to model the progression of periodontal diseases, which involve multiple stages and complex interactions between host tissues and the oral microbiome [16,31]. This systematic review also has limitations. The available evidence is based on a few in vitro studies with potential purposes in clinical practice. Moreover, all the proposed mechanisms had different purposes, designs, methodologies, and outcomes that show their great heterogeneity, making it difficult to carry out more complex quantitative analyses. However, the included studies presented a low level of risk and have a plausible application in periodontal diseases.

## 5. Conclusions

Organ-on-a-chip technology has the potential to advance our understanding of periodontal diseases by providing a more accurate representation of the oral microenvironment and enabling the development of new treatments. Here are some potential uses of organs on a chip in periodontal diseases. They can be used to (1) model the complex interactions between the oral microbiome and host tissues, which can contribute to the development of periodontal diseases, (2) to study the interactions between host cells and periodontal pathogens, and (3) to test the efficacy and safety of potential therapeutics for periodontal diseases. Moreover, they have the potential to be used in personalized medicine by enabling the testing of individualized treatments based on a patient’s unique oral microbiome and host response. These advancements can lead to more effective treatments, reduced reliance on animal testing, and improved overall oral health outcomes, thereby positively influencing public health in the context of periodontal diseases.

## Figures and Tables

**Table 1:** Main features of the included studies.

Authors, Publication Date	Application of the Organ on a Chip	Materials Used	Chip Design	Cell, Tissues/Microorganisms	Culture	Main Results
Makkar et al. 2023 [16]	Gingival connective tissue equivalent cultured on a microfluidic substrate called “gingival crevice-on-chip” under dynamic interstitial fluid flow that mimics gingival crevicular fluid.	Polydimethylsiloxane, an optically clear, flexible material, is used to create the microfluidic device.	Parallel chamber	A human gingival crevice-on-chip microfluidic system was created to allow the cultivation of gingival fibroblasts embedded within a 3D extracellular matrix in the presence of interstitial fluid flow that is physiologically appropriate and unidirectional, simulating the fluid flow in gingival crevices, utilizing live commensal bacteria, such as *Streptococcus oralis*, live pathogenic bacteria, such as *Fusobacterium nucleatum*, and a Toll-like receptor-2 (TLR-2) agonist. A model of the healthy and inflamed states of the connective tissue wall of the gingival crevice was developed utilizing the gingival crevice-on-chip.	To recreate the healthy state of the gingival crevice, which is defined by the colonization of commensal bacteria, long-term bacterial co-culture and colonization investigations were carried out. The crevicular tube was coated with human saliva overnight and then filled with oral commensal bacteria, *Streptococcus oralis*, to recreate the microbial colonization.	It is possible to replicate microbial colonization, the development of biofilm-like structures at the tissue-microbiome interface, long-term co-culture, and bacterial clearance because of simulating gingival crevicular fluid flow utilizing oral symbiont (*Streptococcus oralis*) on a chip. Furthermore, on-chip exposure of the gingival connective tissue analog to the periodontal pathogen *Fusobacterium nucleatum* or the Toll-like receptor-2 (TLR-2) agonist has the capacity to simulate early gingival inflammation. The protective impact of gingival crevicular fluid flow is demonstrated by the generation of simulated gingival crevicular fluid flow toward the bacterial front, which reduces the secretion of inflammatory mediators in contrast to direct exposure.
Jin et al. 2022 [17]	The in vivo gingival epithelial barrier with an immunological micro-environment was closely mimicked by the development of a microfluidic epithelium–capillary barrier with a thin culture membrane.	Negative photoresist and polydimethylsiloxane were used to create the upper and lower layers of the microfluidic device in accordance with conventional soft lithography and microfabrication techniques.	Parallel chamber	Surgery was used to remove one to two mm of healthy gingival tissue from patients between the ages of 18 and 30. The lamina propria and epithelium were divided. The tissue fragments and cell pellets were then gathered and re-suspended in keratinocyte growth media. In the endothelial basal medium, human vascular endothelial cells were grown.	The epithelial and endothelial cell suspensions were seeded on the upper and lower sides of the porous membrane in the device, respectively, to establish a bilayer epithelial capillary on the chip. The sterilized chip’s chambers were filled with liquid and left in a culture medium for a whole night before being inoculated with cells. Human vascular endothelial cells were then added to the chip’s lower chamber after it had been turned over so that it could face upwards. The chip was turned over once more so that its upper chamber faced upward, and human gingival epithelial cells were injected into it after resting for two hours to allow cells to attach.	This periodontal soft tissue device was demonstrated to be capable of simulating the inflammatory process induced by lipopolysaccharides or tumor necrosis factor-alpha in major periodontal soft tissue cell lines, while assessing multiple biomarkers of each periodontal soft tissue cell line, to comprehend the intercellular communication between one another. This in vitro epithelium–capillary interface microarray system may be used as a platform to study how drugs affect the health and function of periodontal soft tissues.
Jalali et al. 2021 [36]	The dynamic interactions between *Streptococcus* species, *Staphylococcus aureus*, and *Actinomyces* species were evaluated, and a microfluidic-based co-culture system and time-lapse imaging were used.	On four-inch wafers, devices were created using conventional soft-lithography methods. Through a photolithography mask, the photoresist was spin-coated onto a silicon wafer and subjected to ultraviolet light. In a 10:1 mixture with a cross-linking agent, polydimethylsiloxane was applied to wafers.	Multiarray	Three species of *Actinomycetaceae* were co-loaded in combination with four species of *Streptococcus*—one strain of *Streptococcus salivarius*, three strains of *Streptococcus mitis*, two strains of *Streptococcus oralis*, and one strain of *Streptococcus cristatus*—to study the co-culture characteristics of *Actinomycetaceae* and *Streptococcal* species in the microfluidic chambers.	On Chacolate II agar, *Actinomyces graevenitzii* was cultivated at 37 °C in an anaerobic incubator. Overnight incubation of *Streptococcus cristatus* was performed at 37 °C with shaking. Hemocytometer measurements of bacterial suspension concentrations were used to alter the final bacterial concentration, which was then diluted in Iscove’s Modified Dulbecco’s Medium with 20% fetal bovine serum. Infusion agar plates made from the brain and heart were frequently used to produce bacterial cultures. It was used in nanoliter co-cultures and microfluidic devices.	When *Streptococcus cristatus* or *Streptococcus salivarius* were co-cultured in nanoliter containers with *Actinomyces graevenitzii*, it was discovered that these bacteria were specifically excluded from the media around the microcolonies of *A. graevenitzii*. This community structure did not exist in co-cultures containing other *Actinomycetaceae* species such as *Streptococcus odontolyticus* or *Actinomyces naeslundii* or with *Streptococcus mitis*, *Streptococcus oralis* or isolates of those species. Additionally, compartments containing both *A. graevenitzii* and *Staphylococcus aureus* drew fewer neutrophils than compartments having an equivalent amount of either species alone, indicating a potential survival advantage when immune responses are combined.
Kristensen et al. 2020 [37]	It was shown how to create a brand-new, adaptable flow cell for microscopy that can handle samples of various geometries and offers shear-controlled flow. The flow cell is created using specialized 3D software, which makes it simple to alter the geometry to fit the given sample.	A flow cell with a customizable shape was created for microscopy examination of samples of in situ-grown biofilms in shear-controlled flow. The flow cells were made as single-piece disposable models, printed in three dimensions from resin, and sealed with a coverslip after the biofilm sample was inserted.	One chamber	The flow cell was used to monitor pH variations in various microenvironments within intraorally developed biofilms from one participant on glass slabs.	On the days of biofilm collection, the participant submitted paraffin-stimulated saliva samples to obtain a realistic flow medium. The saliva samples were cleaned by centrifugation (5 min, 1150 g), filtered through sterile gauze, and then utilized instantly. The biofilms were exposed to a flow rate of 5 mm/min, which corresponds to stimulated saliva flow in the oral cavity, after 30 min of static incubation.	pH decreased in the biofilms under static conditions, with noticeable variations between individual biofilms as well as between various microscopic fields of view inside a single biofilm. The pH of the biofilms’ top layer tended to be lower than that of their bottom layer. The pH of all biofilms increased to neutral or slightly alkaline values when saliva flow was induced, and the vertical gradients were reversed, with the bottom of the biofilms being more acidic than the top. The significance of flow for the investigation of pH in dental biofilms was established.
Luo et al. 2019 [38]	A microfluidic biofilm system-based image analysis tool called Biofilm Architecture Inference for quantifying the architecture of oral multi-species biofilms after anti-biofilm interventions was tested. Architectures of treated and untreated biofilms were contrasted, as well as those treated with sodium gluconate (placebo), stannous fluoride, and water (negative control).	Biofilm Architecture Inference Tool, a piece of software that quickly analyzes the structure of biofilms photographed using a confocal laser scanning microscope, was introduced. The program combines plug-and-play automation with a graphical user interface to give users a simple way to examine the architecture of biofilms.	One chamber	At least five healthy, non-smoking individuals’ saliva was collected in batches. Saliva samples were pooled. Both cell-free saliva and cell-containing saliva (the inoculum and growth media, respectively) were prepared.	On 24-well Bioflux plates, a 1 mL sample of saliva-containing cells was added to the outlet wells. The surface of the plate was then treated for 20 min at room temperature. Each treatment well-received additions of various stannous fluoride treatment or placebo treatment concentrations. To imitate higher shear force brought on by brushing or swishing, the treatment schedule was set at 2.0 dynes/cm^2^ for 2 min. Imaging was performed at five points evenly spaced across the viewing port to record the growth of the biofilm.	Biofilm biovolume, the total number of objects, surface area, fluffiness, connectedness, convex hull porosity, and viability were all calculated using the Biofilm Architecture Inference Tool. Image analysis revealed that 3439 and 10,000 p.p.m. dramatically changed the architecture of the oral biofilm. Treatment with stannous fluoride reduced biovolume, surface area, object count, and connectedness while increasing fluffiness (*p* < 0.01). The ability of the Biofilm Architecture Inference Tool to evaluate changes in biofilm architecture and identify potential antimicrobial and anti-biofilm effects of candidate drugs was demonstrated.
Rahimi et al. 2018 [39]	Mucosal remodeling and the reactions of epithelial and subepithelial layers to stressors frequently seen in the oral environment were assessed. This study set out to create a co-cultured microfluidic mucosal model on a chip.	Eight sets of microchannels were present on each chip. There are six polydimethylsiloxane posts and seven square pores between each set’s three channels. Custom microfluidic device molds were made utilizing conventional photolithography, a spin coater, and an exposure-masking system on a 4 in. silicon wafer.	Parallel chamber	For the mucosa-on-a-chip, two immortalized human cell lines were grown. In polystyrene tissue culture flasks covered in type I collagen, the keratinocyte cell line and human gingival fibroblast cell line were kept alive.	Keratinocytes and fibroblasts were cultured in Prigrow III and IV medium, respectively. A steady co-culture construct was created when human gingival keratinocyte and fibroblast cell lines were added to a 3-channel microfluidic device.	The creation of an oral mucosa-on-a-chip resulted from the formation of a submucosal layer of fibroblasts inserted in collagen in a central conduit, followed by trans-channel planting of keratinocytes into pores between polydimethylsiloxane posts. This structure enabled convenient and accurate tracking of keratinocytes and fibroblasts using conventional microscopy, with posts permitting for dual attachment of keratinocytes to apical and basal surfaces, as appears in the junctional epithelium. After 2-hydroxylethyl methacrylate exposure, cell viability decreased, and transepithelial electrical resistance declined following *Streptococcus mutans* culture from the luminal channel. These findings establish a microfluidic culture-on-a-chip methodology to test the oral mucosa hypothesis.
Lam et al. 2016 [40]	A high-throughput microfluidic “artificial teeth” device was described that offered controls of various microenvironmental elements (such as nutrients, growth factors, dissolved gases, and seeded cell populations) for quantitative traits of long-term dental bacterial growth and biofilm development.	From top to bottom, the microfluidic device was constructed using a multilayer soft lithography of polydimethylsiloxane for the microchannel layers: gas control channels, oxygenation channels, water jackets, flow channels, and culture chambers.	Multiarray	The biofilm samples were taken from a human oral cavity. Based on their prevalence and significance in the formation of the dental biofilm, the target species were selected to be *Sprepptococci*, *Fusobacterium nucleatum*, *Actinomyces naeslundii*, and *Porphyromonas gingivalis.*	Dental bacteria samples were parallel cultured under four daily dissolved oxygen cycles to show the impact of the dissolved oxygen profiles on the bacterial species contents in the dental biofilm. A healthy pH buffer and the nutritional equivalent of saliva is basal medium mucin. A basal medium recipe was used to create it. A solution containing 5 g/L trypticase peptone, 10 g/L proteose peptone, 5 g/L yeast extract, 2.5 g/L potassium chloride, 5 mg/L hemin, 1 mg/L menadione, 2.5 g/L gastric mucin, 1 mmol/L urea, and 1 mmol/L L-arginine was used to create a basal medium mucin.	As the dental biofilms developed, the device for artificial teeth was able to evaluate the biofilm’s form, colonization density, and spatial arrangement of bacterial species. By measuring the cluster thickness, cell viability distribution, and percentage of the major dental bacteria (*Streptococci*, *Fusobacterium nucleatum*, *Actinomyces naeslundii*, and *Porphyromonas gingivalis*), this device may acquire quantitative biofilm characteristics. Moreover, the multiplexed platform’s scalable architecture allowed for the monitoring of several culture samples that were exposed to various environmental conditions, which reduced the considerable experimental efforts required in traditional laboratory settings.

**Table 2:** Risk of bias of the assessed studies.

Study	Design Objective Sample Baseline Characteristics Co-Interventions	Measures Measurement Method Blinding Examiner Blinding Statistician Described Reliability. Level of Agreement	Analysis Statistical Appropriate Analysis Co-Interventions Subgroup Analysis Statistical Significance Confidence Intervals	Clinical Significance	Total
Makkar et al. 2023 [16]	4	3	4	1	12
Jin et al. 2022 [17]	4	3	4	1	12
Jalali et al. 2021 [36]	4	3	5	1	13
Kristensen et al. 2020 [37]	4	3	4	1	12
Luo et al. 2019 [38]	4	3	4	1	12
Rahimi et al. 2018 [39]	4	3	4	1	12
Lam et al. 2016 [40]	4	3	4	1	12

## Data Availability

The data obtained in this review were pooled from the included investigations.
